# Understanding the Role of Fibroblasts following a 3D Tumoroid Implantation for Breast Tumor Formation

**DOI:** 10.3390/bioengineering8110163

**Published:** 2021-10-27

**Authors:** Girdhari Rijal

**Affiliations:** Department of Medical Laboratory Sciences and Public Health, Tarleton State University, a Member of Texas A & M University System, Fort Worth, TX 76104, USA; rijal@tarleton.edu

**Keywords:** fibroblasts, cancer-associated fibroblasts, tumor growth, tumoroids

## Abstract

An understanding of the participation and modulation of fibroblasts during tumor formation and growth is still unclear. Among many speculates, one might be the technical challenge to reveal the versatile function of fibroblasts in tissue complexity, and another is the dynamics in tissue physiology and cell activity. The histology of most solid tumors shows a predominant presence of fibroblasts, suggesting that tumor cells recruit fibroblasts for breast tumor growth. In this review paper, therefore, the migration, activation, differentiation, secretion, and signaling systems that are associated with fibroblasts and cancer-associated fibroblasts (CAFs) after implantation of a breast tumoroid, i.e., a lab-generated tumor tissue into an animal, are discussed.

## 1. Introduction

The traditional two-dimensional (2D) in vitro culture has been in practice for more than decades to perform fundamental cancer research. Though it is still very popular, it cannot support breast tumor growth by maintaining physiological conditions with tumor heterogeneity. It is, therefore, not more supportive of revealing tumor morphology and tumor clonal heterogeneity. Recent advances in 3D culture systems have provided insight into a long-standing desire for understanding tumor growths and rapid diagnosis of cancers together with effective treatment plans [1]. They have optimized and applied alternative systems for maintaining the tumor heterogeneity where cells express phenotypical and physiological characteristics similar to their expressions in native tumors [2]. Tumoroids, organoids, and 3D-cell laden scaffolds are some of alternative systems that have been applying to generate mini tumor-like tissue in the lab [2,3,4,5,6,7]. They are multicellular with one or more different types of cancer cells, aggregating in a manner to exhibit the physiological relevant cell–cell and cell–extracellular matrix (ECM) interactions. Tumoroids somehow resemble natural tumors according to certain characteristics they express, for example, cell signatures, heterogeneity, and structural complexity. Their intermediate complexity that lies between standard 2D cultures and native tumors further facilitates studying tumor growth in a lab. It is said that tumoroids can maintain the stroma of in vitro tumor tissue, representing native tumor biology. A tumoroid is a type of transplantable tumor, which is easy to generate in a lab, and can be optimized according to study projects.

Further, growing tumoroids can be monitored for various functional studies of cytokines for ECM remodeling, proliferation, migration, cell–cell interaction, and many more, through state-of-the-art live and post-live imaging systems [8,9,10]. They have to be optimized to address some other concerns, though current tumoroid models provide many clues about tumor tissue formation, for example, how the tissue fibroblasts migrate and take part in tumor formation. It is difficult to reveal the fundamental process of tumor tissue formation from pro-oncogene or migrating cancer cells by using imaging systems because of tissue complexity and complex cell signatures in a native tumor [11,12,13]. Therefore, an implanted tumoroid can represent a tumor core and can facilitate breast tumor formation by triggering migration of cancer-associated cells, including fibroblasts towards it [14,15,16]. For a more concise and informative review, here, only fibrocytes and fibroblasts are described, not other cell types, even though they engage in tumor formation. A tumoroid is a lab-grown tumor of a desired size. After implantation, it forms a xenografted tumor in an animal tissue within a certain period, depending on the type of cancer cell and hydrogel or scaffold used [4,7,16,17]. Fibroblasts and fibrocytes are the most dominant cell types in a tumor tissue, more significant than the cancer cells and other cell types, as shown in various studies in the literature [18,19,20].

**Figure 1:**
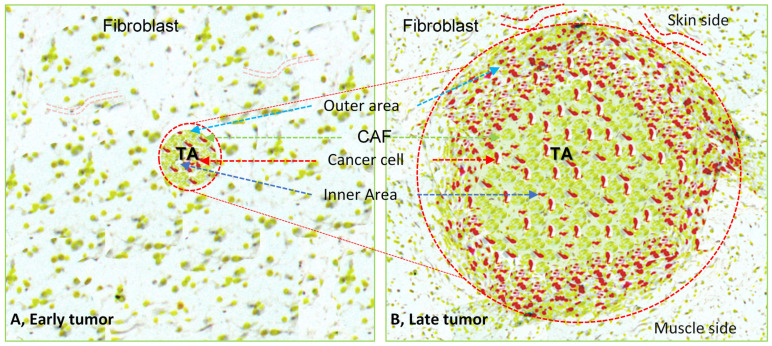
Illustration represents a xenografted tumoroid at an early period and giant solid tumor tissue at a later stage, surrounded by host tissue. The micro-tumoroid generated in the lab has implanted into the subcutaneous tissue of the recipient animal (**A**). Local immune cells or inflammatory cells act on the tumoroids, triggering chemotaxis that enhances the migration of the different host cells to the implanted tumoroid area to develop the large solid tumor (**B**). The histological section of the large tumor shows its central core has round fibroblasts (yellowish green color) with scattered cancer cells (red color), tumor margins with more active tumor cells (red color) and cancer-associated fibroblasts (elongated yellowish green color) along with other host cells (not shown). There are more blood vessels on the skin side of the tumor than on its muscle side when the tumoroid transforms to a xenografted tumor into the subcutaneous pouch of the recipient animal. TA, tumor area, encircled by red dotted line.

**Figure 2:**
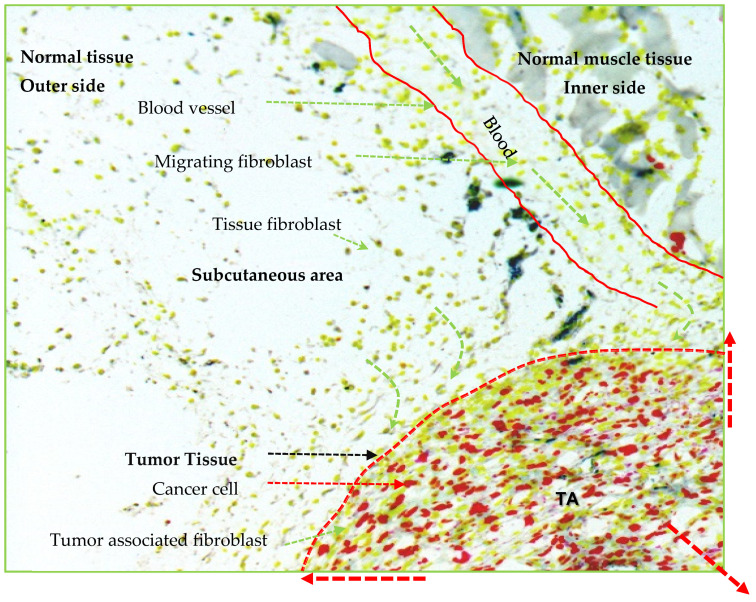
Illustration represents growing tumor in three dimensional. The local tissue fibroblasts (yellowish green color) predominantly participate in tumor expansion after getting signals from cancer cells (red color) and converting to the activated cancer-associated fibroblasts (CAFs). Circulatory fibroblast or fibrocyte through small or large blood vessels also participate in tumor expansion though less as compared with the local tissue fibroblasts. The tumor tissue area (marked by red dotted margin) represents the outer layer of growing tumor that contains the rapidly proliferating cancer cells (red color) and CAFs (elongated yellowish green color). Note: Other cells are not represented in the figure even if they participate in tumor formation. TA, tumor area.

Histological sections of a tumor have consistently shown that both tissue fibroblasts and migrating fibroblasts or fibrocytes take part in the tumor tissue growth, as shown in the Figure 1 and Figure 2 [16,18,19]. Fully grown tumor tissue has a large inner core area that is surrounded by the outgrowing tumor tissue. Interestingly, active or proliferating cancer cells are present predominantly in the outer margin of tumor tissue [16,20,21], while cancer-associated fibroblasts (CAFs) primarily occupy the core part rather than the outer growing tumor region [17,18,19,20]. In addition, the percentage ratio of CAFs to cancer cells is significantly more towards the core region, with a reversed ratio in the outer region of a tumor tissue, as represented by the Figure 2 [4,16,22,23]. On the one hand, the cancer core, because of less blood and nutrition supply, is usually the detective core which represents the positive charge topological area that is occupied mostly by round fibroblasts. The outer part of a tumor, on the other hand, represents a negative topological charge area which consists mostly of elongated fibroblasts that are necessary for tumor growth, as shown in Figure 2 [21]. Kristen et al. effectively described the cellular changes by topological charge, for example, degree of alignment, as well as behavior and density of fibroblasts and epithelial cells [24]. Fibroblasts tend to be more alignment with more density, but they have different behaviors near a defect as compared with normal tissue [24]. Because of heterogeneity and the versatile phenotypic nature of cells in a tumor tissue along with functional plasticity of CAFs, a detailed study on fibroblasts and CAFs could facilitate the understanding of tumor biology in a xenografted tumor [25].

## 2. Fibroblast Contribution to Tumor Tissue Formation

### 2.1. Tissue Fibroblasts

Most of the cell components of a tissue are fibrocytes, which are usually in a quiescent state. They transform to an active state known as quiescent normal fibroblasts when tissue maintenance and metabolism are necessary to maintain the homeostasis. They also play a vital role in intra- or inter-cell communications to regulate a normal tissue microenvironment, in addition to their support in the immune system at the tissue level (more information in paragraph 2 of Section 3.6) [22,26]. The relatively high expression of CD39 usually differentiates fibrocytes from quiescent normal fibroblasts [23,27]. The reversible conversion of fibroblasts to fibrocytes takes place at a cellular level, as per the need to maintain the cellular integrity and tissue function [27,28]. Fibroblasts change their phenotypical features and physiological functions, depending on purposes and action sites. Interestingly, fibroblasts remember their original locality and functions even after their translocation to other body site [29,30,31,32]. They are primarily derivatives of primitive mesenchyme from epithelial cells through epithelial-mesenchymal transition (EMT) [33].

Fibroblasts handle the connective tissue structural integrity, and release various ECM precursors required for formation of tissue matrix, a fundamental foundation for tissue development and differentiation [34]. Interestingly, fibroblasts undergo mesenchymal-epithelial transition (MET) when there is a requirement of producing epithelia which is necessary for the regeneration of both normal and abnormal (for example, tumor) tissues [35]. Fibroblasts are heterogeneity in population depending on the tissue source, activation, and function. Many studies mention that local tissue fibroblasts predominantly transform into CAFs in a tumor microenvironment as compared with circulating fibroblasts [36]. A histological evaluation usually shows the morphological diversity and variability of fibroblasts in addition to their distribution patterns within solid tumors [37]. Busch et al. effectively presented the heterogeneity of fibroblasts at the molecular level based on an analysis of both a single fibroblast and its tumor-activated counterpart [25]. They showed that most fibroblasts representing the “naïve” phenotypes expressed the typical fibroblast markers (e.g., VIM and CD44) without the expression of epithelial markers (e.g., CDHA and EPCAM) [25]. Various factors produced by the tissue fibroblasts are mostly cell specific, not species specific, and their release depends mostly on the fibroblasts themselves. They can modulate the epithelial cell mobilities, and usually work as paracrine agents for fibroblast–epithelial cell interactions [38]. These factors also act in a group as a restrictive effect, a specialized contractile machinery necessary for motility which is applied on intercellular matrix materials, enforcing fibroblasts to form aggregates for migration [39]. Fibroblasts, which have round cell morphology, have higher density with positive topological charges and they are present in a defect area, whereas fibroblasts having elongated morphology with negative topological charges and occupy the growing tissue area. The distribution pattern of different morphological fibroblasts suggests the possibility of cell alignment during tissue growth. The elastic nature of tissue further supports cell alignment and facilitates the precise location of an oriental core defect. It also guides the migration and the differentiation of fibroblasts in addition to apoptosis [21].

### 2.2. Migrating Fibroblasts

**Figure 3:**
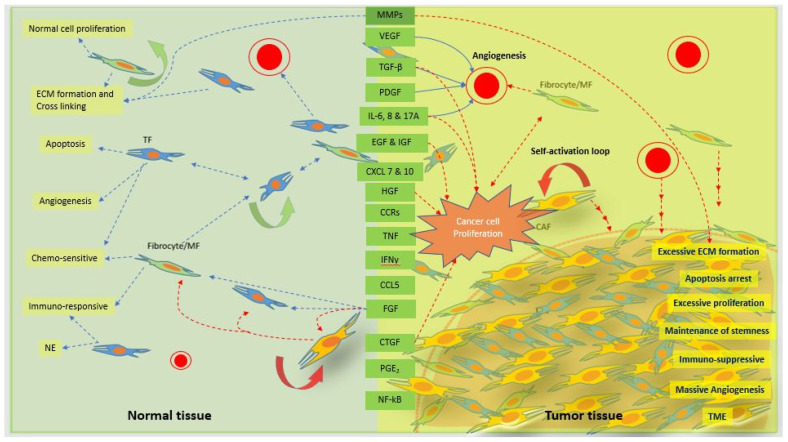
Schematic representation of the signaling cascades of the normal fibroblasts and CAFs for normal and tumor tissue function, respectively. Normal tissue fibroblasts and/or myofibroblast (MF) or fibrocytes play a vital role by maintaining normal tissue homeostasis through normal tissue proliferation, ECM secretion, cell–cell and cell–ECM interactions, angiogenesis, apoptosis, and chemosensitivity through various inter- and intra-coordinated communications. Normal communications within normal cells through various signaling factors and cascades are disturbed and disoriented by cancer cells, leading to excessive cell proliferation and ECM secretion. ECM becomes stiffer with a significant increase in inter- and intra-cell to cell, or cell to ECM communications. Tumor tissue has a massive angiogenesis process with the ability to suppress apoptosis mechanisms. Increased tolerance by tumor tissue to an anticancer therapy speeds up uncontrolled tumor tissue growth as compared with check-and-balance normal tissue growth. The biological factors in the green box represent the factors released from both normal fibroblasts at a normal level and CAFs at an excessive level. The dotted blue arrows represent the normal secretion of factors and function on different cells for normal homeostasis, and the dotted red arrows represent the excessive secretion of factors and function on different cells for tumor tissue microenvironment. MMPs, matrix metalloproteinases; VEGF, vascular endothelial growth factor; PDGF, platelet derived growth factor; IL-6, interleukin-6; IL-17A, interleukin-17A; EGF, epidermal growth factor; IGF, insulin growth factor; CXCL 7 and 10 platelet-derived chemokines CXC chemokine ligand (CXCL) 7 and 10; HGF, hepatocyte growth factor; CCR, C-C chemokine receptor types 2; TNF, tissue necrotic factor; INF-γ, interferon-γ; CCL5, C-C motif chemokine ligand 5; FGF, fibroblast growth factor; CTGF, connective tissue growth factor; PGE2, prostaglandin E2; NF-kB, nuclear factor kappa-light chain. Self-activation property of CAFs supports for rapid tumor cell proliferations and tumor growth. TF, tissue fibroblast; NE, normal environment; TME, tumor microenvironment.

Local tissue migration is possible by micro-level movement, and distant migration is by macro-level movement, of fibroblasts through blood. Circular fibrocytes or fibroblasts are distant migrating fibrocytes or fibroblasts. Fibroblasts should polarize spatially through the direction of various signaling molecules to achieve migration ability and to promote cellular protrusion at one end while retraction at the other [40]. A leading or protruding edge periodically switches the protrusion and retraction process, depending on the fluctuation in actin polymerization that is controlled by complex motility dynamics signals [41,42]. Cell migration depends on ECM rigidity, fibronectin binding capacity, and myosin light chain kinase (MLCK) that exhibit the control on Rac signaling and periodic protrusion. Rac signaling controls the protrusion width, thereby, increasing the mobility of a migrating cell (Figure 3 normal tissue) [42]. The migrating cell can change the orientation and direction by restricting the protrusion in the opposite direction through a stochastic turning mechanism, which is directed by various chemotactic gradients and other dynamic cues (Figure 3 tumor tissue) [43]. It has been shown that PIK signaling mediates for the establishment of chemotactic gradients and chemotaxis and also restricts for additional protrusion, facilitating unidirectional cell migration [44,45,46]. Blood-borne fibroblasts-like cells have a peculiarity in their cell phenotypes (collagen I/CD11b/CD13/CD127/CD45RO/MHC class, II/CD86/TSLPR, and alpha-SMA) [47,48], and take part actively in tumor formation [49]. They migrate to the tumor formation site through specific communication among cytokines and their receptors, for example, CCR3, -5, -7, and CXCR4 (Figure 3) [48]. TGF-β plays a vital role in proliferation and differentiation of fibrocytes, and enhances the production of ECM proteins, for example, collagen (Figure 3) [50]. In addition, it mediates the fibroblast migration with the support of CD44 [21]. CD44 is a cellular adhesion receptor that is usually expressed during tissue inflammation and injury [51]. Its interactions with cytoskeletal components influence the adhesion and motility of fibroblasts for tissue remodeling and repairing [52]. It further promotes TGF-β activation through MMP molecules, enhancing fibroblast migration, and therefore speeding up migration velocity to spike cellular activities to maintain tissue integrity [52]. In addition, activated T cells that interact with migrating fibroblasts help early differentiation and maturation of fibroblasts, and support fibroblasts to take part in different functions, for example, promoting tumor stromagenesis [53]. Migrating fibroblasts serve as the regulators of cell migration and also directly participate in cancer progression, as shown in Figure 3 [49]. They also promote the influx of monocytes into the tissue by increasing the expression of certain receptors (CCR2 and CCR5) (Figure 3), which speed up the vulnerability of the cancer cell invasion [48,49]. In addition, they mediate the immune suppression in tumor tissue by increasing the expression of indoleamine oxidase, and therefore they are also considered to be immunosuppressive fibroblasts. Circulatory fibroblasts also help to induce angiogenesis during tissue repair or tumor tissue growth (Figure 3) [48]. 

### 2.3. Activated Fibroblasts

Activated fibroblasts take part more actively in various functions, for example, production and expression of various proteins or cytokines, and activation of other cells through cell-cell communications directly or indirectly as compared with the normal fibroblasts. They are the most common cell type that produces the collagen, a major component of ECM proteins, and other protein factors or fibers. Quiescent fibroblasts become active sufficiently only for a normal tissue repair process so as to maintain tissue integrity, in the case of tissue that becomes damaged by various reasons. Normally, an injury of tissue triggers an inflammatory response, thereby, chemoattracting inflammatory cells that secret various factors to support the transformation of local fibroblasts to activated fibroblasts [54]. Activated fibroblasts usually locate in any inflammatory site to repair the tissue damage by depositing tissue matrix, and to support angiogenesis [55,56]. They become active exceeding the normal limit, for example, during abnormal tissue growth by the signals from abnormal sources or cancer cells [57,58]. Normally activated fibroblasts represent the normal or negligible expression of proteins or cytokines, for example, normal expression of Col-I without alpha-SMA expression as shown by dermal fibroblasts. However, in the presence of cancer cells, dermal fibroblasts express abnormally high Col-I and alpha-SMA [36,59]. Activated fibroblasts mainly maintain tissue homeostasis by performing various required functions, for example, ECM production and modification, tissue regeneration, angiogenesis, immunity, chemo-sensitivity or chemo-resistance, and cell metabolism, along with program cell death (apoptosis). However, almost all the expressions by super-activated fibroblasts with the apoptosis arrest are abnormally high in tumors. Fibroblasts become excessively active in a tumor microenvironment, and support breast tumor growth through versatile functions of many secretory proteins, cytokines, and transcriptional factors, for example, MMPs, VEGF, TGF-β, PDGF, IL-6, EGF, IGF, IGF, FGF, CTGF, PGE2, CXCLs, CCRs, CCLs, TNF, INF-γ, and NF-kB, etc. (Figure 3) [60,61,62,63,64,65].

### 2.4. Fibroblasts in Tumor Mass

The recruitment and activation of fibroblasts in growing tumors are the complex processes to figure out because of tissue complexity, heterogeneity, and the dynamic tumorigenesis. In a solid tumor mass, stroma includes the fibroblasts, blood vessels, inflammatory or immune cells, fat cells, and cancer cells where cancer cells exhibit guidance to co-ordinate cellular flow and migration [66]. It has been shown that circulatory fibroblasts do not contribute significantly for their differentiation to CAFs as compared with local tissue fibroblasts. Circulatory fibroblasts help immune influx to the tissue, facilitating invasion by tumor cells. In addition, CAFs may be originated from adipocytes, or even from cancer cells via EMT [67]. CAFs, which express Col-I, constitute approximately one-third of the stromal mass [36]. They also promote the vessel sprouting for the development of the tumor vasculature or angiogenesis [36,68]. Their support in EMT transition leads to tumor stemness, and drug resistance as mentioned above (Figure 2) [58,69,70,71]. CAFs reprogram the regulatory molecules, and change the metabolic pathways to favor the tumor growth (Figure 3) [23]. They not only represent the predominant cell component in the tumor but also secrete the major extracellular proteins or fibers that are needed for the formation of tumor stroma (Figure 2 and Figure 3). 

## 3. Other Factors That Contribute to Tumor Tissue Formation

### 3.1. ECMs

ECM is a conditioned structural framework created by cells for their attachment, proliferation and differentiation. Cell-to-matrix interactions are prerequisite for regulating normal tissue homeostasis. The change in regular ECM integrity considerably influences the activity of fibroblasts, for example, attachment, proliferation and secretion [60]. Likewise, changes in the cellular expressions alter the ECM structure and integrity [60,61]. CAFs usually express markers like CD140b, CD87 and CD95 that help to differentiate themselves from the normal activated fibroblasts [23]. Unusually high expression of these markers contributes to tissue disorganization directing for the aberrant tissue growth as noticed in an unhealed wound or in a cancer. In both unhealed wound and cancer, ECM becomes stiffer compared to surrounding normal tissue ECM [72]. The CAFs and local fibroblasts activated by tissue inflammation [72,73] induce excessive production, remodeling and stiffening of ECMs. Newly deposited ECM further supports for the reorientation of collagens and other ECM fibers by the cross-linking through LOX and transglutaminase, generating larger and stiffer ECM fibers along with enlargement of the tumor tissue [72,73,74]. The CAFs from patient-derived breast cancer have shown the moderate secretory profile on molecular signatures with most ECM remodeling molecules, for example, COL1A, TNC, MMP2, LOX and LOXL2 [25]. It might be the reason that CAFs-derived ECM proteins are more progressive towards the stiffness and promote for the cancer growth and invasion [71]. Detail description about the changes in ECM structure and integrity during migration, initiation and formation of tumor by fibroblasts is nicely described by Tianyi et al. [71].

### 3.2. Angiogenesis

Angiogenesis is a complex and multistep process that leads to the development of new blood vessels through various co-ordination systems either during normal tissue development, wound repair or during cancer growth [64]. Fibroblast growth factor 2 (FGF2) of FGF family is a potent angiogenic factor that induces endothelial cell proliferation, migration and initiation of tubule formation along with the promotion of various other enzymes like proteases, and supports for the receptor expressions like integrin and cadherin [62,63]. FGF2 inhibits TGF-β1, inducing the differentiation of fibroblasts for pro-fibrotic microenvironment, and enhances proliferation of fibroblasts for new tissue formation and angiogenesis [75]. Further, FGF supports vessel integrity, stimulates the production of vascular endothelial growth factors (VEGFs), and controls the glycolytic metabolism in endothelial cells that are needed for the angiogenesis process. Some studies have shown that lack of FGF effects angiogenesis because of failure in vessel integrity, and the increase in permeability [65,76,77]. As known, CAFs speed up the angiogenesis in cancer tissue through VEGF-mediated enhancement of zester2/vasohibin 1 (EZH2/VASH1) pathway [78]. 

In addition to support for angiogenesis by angiogenetic factors, fibroblasts directly participate in formation of outermost layer of blood vessels, especially of large blood vessels, generally known as adventitial fibroblasts [79]. They possibly play a role as vascular progenitor cells of unknown heterogeneity. These distinct niches of perivascular fibroblasts share some expression markers with vascular smooth muscle cells and pericytes, creating the strong lineation that makes difficult to delineate them from underlying layer [79]. Arsheen et al. studied about the various collagen genes expressed by perivascular fibroblasts to establish the vascular integrity [80]. Interestingly, perivascular fibroblasts also function as pericyte progenitors to maintain the inner layer of the blood vessel and deposit matrix proteins to form the endothelial lumen and to stabilize nascent blood vessels [56,80]. CAFs additionally support vascular growth through mechanical force created by the increase in the fibrin density [81].

### 3.3. Immunosuppression 

Besides support for tissue maintenance and angiogenesis, activated fibroblasts secret various peptide growth factors, chemokines, cytokines and so on to regulate the immune status within the tissue microenvironment (Figure 3). Since the expression profile depends on the location and the function of fibroblasts, it is unclear to describe the role of fibroblasts precisely in immunosuppression. But some studies show that fibroblasts suppress the immune system through multi-step mechanism, supporting for the tumor cell survival and the cancer growth [71,82]. CAFs however support accumulation of inflammatory cells and modulate immune response through TGF-β besides dysregulation of the ECMs [83,84,85]. They mediate the higher expression of CD206, an anti-inflammatory molecule, showing the immunosuppression activity indirectly [85]. They are derivatives of different cell types such as normal fibroblasts, epithelial cells, bone marrow stromal cells, stellate cells and adipocyte cells [86]. CAFs communicate and stimulate the self-renewal function of cancer cells mainly through the paracrine systems facilitated by cytokines, vesicles and metabolites (Figure 3) [86]. They are responsible not only for excessive production of ECMs but for their physical remodulation, supporting proliferation and migration of cancer cells [86]. CAFs also play a vital role to protect cancer cells in tumor microenvironment indirectly by secreting MMPs, upregulating BCL-Xl, and by releasing some soluble factors, for example, fibroblast activation protein [87,88,89,90,91,92,93]. Further, CAFs derived exosomes, which establish the intercellular communication, induce fibroblast differentiation to CAFs through TGF-β signaling, and promotes the chemoresistance [94,95].

### 3.4. Energy

Survivability and activities of fibroblasts depend solely upon energy and energy sources available in surrounding microenvironment. Various activities of fibroblasts like self-activation, production of ECMs, and regulation of other processes, for example, angiogenesis require energy. Fibroblasts acquire energy only through the metabolism of carbohydrate, lipid or proteins. Fibroblast growth factors like FGF1, FGF15, 19 & 21 have emerged as key regulators of carbohydrate and lipid metabolism [96,97]. Fibroblasts also can mobilize tumor cell glycogen to promote the cancer cell proliferation [98]. In addition, TGF-β1 produced by cancer cells activates p38-MAPK signaling in CAFs to release various chemokines and cytokines so as to help for the mobilization of glycogen within cancer cells [98]. CAF associated FAK can regulate expression of some chemokines like Cc16 and Cc112, and help metabolism in cancer cell through malignant Ccr1/Ccr2 and PKA activation [99]. Interestingly, both CAFs and cancer cells exhibit a metabolic shift in their biosynthetic pathways which are under the direction of epigenetic reprogramming on lactate production [100].

### 3.5. Apoptosis

Apoptosis represents the natural cell death, and it is necessary for the regulation of tissue homeostasis as well as development and remodeling of a tissue. Fibroblasts regulate apoptosis in normal tissue by the help of various cytokines, chemokines or active protein that can cause glutathione depletion in the cells [101]. ROS-induced cell death may represent, depending on the cellular system, either apoptosis or necrosis. Fibronectin also induces the delayed apoptosis in fibroblasts compared to early apoptosis in endothelial cells [102]. Proliferation of fibroblast and its apoptosis depend on various metabolic activities [103,104], hormonal control [105], and hypoxic condition [106]. In tumor, apoptosis is markedly reduced compared to proliferation of fibroblasts. Fibroblasts therefore not only represent the predominant cell component in tumor but also contribute for the formation of tumor stroma by depositing major secretory proteins and fibers (Figure 2 and Figure 3). 

### 3.6. Tumor Microenvironment

The tumor microenvironment (TME) has distinct ECM proteins and its regulators compared to those of native tissue microenvironment [16]. An in vitro tumoroid prepared from the single cancer-type in the lab exhibits unique ECM proteins and other factors specific to the cancer cell type used in its formation. These ECM proteins and factors are both insoluble and soluble cancer factors and handle the formation of an entire cell-specific TME. The studies have shown that the cancer cells express some ECM components that are not expressed by normal cells, for example, Col19A1, Col122A1, Col7A1, LAMA4, LAMB1, LTBP3, TINAGL1 [107]. Cancer cells secrete the ECM regulators and other factors to maintain the tumor ECMs, for example, LOX, LOXL2, LOXL4 & PLOD1, S100-A4, S100-A6 & S100-A13 [107]. 

The tumoroid with pre-established TME therefore when implanted into the mice, cancer cells do not need to go through the new environmental adapting process to create their initial TME. However, host cells get new tumor mass that triggers the change in cell signatures of host tissue and directs the extension of TME thereby facilitating transformation of the tumoroid into the tumor mass within the host tissue (Figure 3). The first host response to the tumoroid is inflammatory response where immune cells migrate towards the tumoroid and act against the complex tumor tissue antigens present in the tumoroid (tumor ECMs, regulators and secret factors etc.). Local fibroblasts promote the inflammation and induce immune cell influx, forming the space to migrate themselves towards the tumoroid. Ultimately, immune cells become more activated by the signals received from the cancer cells, leading to profuse secretion of ECM proteins and other factors (Figure 3) [108,109,110]. In addition, tumoroid cancer cells adapt in host tissue and proliferate rapidly together with invasion of tumoroid by local immune cells, fibroblasts, endothelial cells, and fat cells, leading tumoroid transformation to a tumor mass (Figure 1, Figure 2 and Figure 3). As shown in Figure 2, fibroblasts occupy the majority of the central core of solid tumor, however, cancer cells and active CAFs mainly occupy the tumor peripheral area [16,21]. 

It is known that the greater the formation of blood vessels in TME, the higher the proliferation of cancer cells and CAFs, thereby, extending the area of tumor mass (more often in a skin site) as compared with an area with less blood supply (more often in a muscle site) where tumor mass cannot extend much (Figure 2) [4,111,112]. The histology and immunohistochemistry of xenografted tumor sections have shown that the outer area of a tumor has an abundant blood supply with many fat cells as compared with the central area of a tumor [4,113]. It suggests that, as the tumor grows, the cells in the central core area are relatively less active or in the quiescent phase or undergo necrosis after a certain period. Later, liquid stroma fills the tumor necrotic core [114,115]. 

The CAFs are one of the vital cell components in a tumor and their self-proliferating loop further increases tumor mass. CAFs enhance the endothelial cell proliferation for more vascularity, protect the tumor cells from anticancer drug effect, increase the stiffness of ECMs, and activate the fatty cell proliferation for the increased energy production required for the highly proliferating fibroblasts themselves, cancer cells, and other associated cells (Figure 3) [58,69,70,71]. Xenografted tumors generated in a host tissue by implanting the cell- and ECM-specific tumoroids, therefore, help decipher the significant roles of fibroblasts for cancer formation and growth.

## 4. Tumoroid Transformation to a Tumor by Host Fibroblasts Following Implantation

Tumoroids are sophisticated and complex 3D spheroids that contain at least one type of cancer cells [15]. A fully grown tumoroid, where a necrotic core is surrounded by actively proliferating layers of cells, resembles native tumor tissue [116]. Studies have shown that cells involved in a tumoroid perform metabolic and physical activities similar to what they do in native tissue. The ECM proteins produced by cells during tumoroid formation bind each other and form the complex networks that represent the tumor complexity as seen in xenografted tumors [117,118]. The methodologies to generate tumoroids are beyond the scope of this paper. Briefly, most of the methods follow the techniques which help to aggregate cells after their division, for example, magnetic levitation [119]. Tumoroids have been used to study the invasiveness of a tumor cell, anticancer drug screening, and various cell signatures [4,120].

Biological mechanisms either in normal or in altered states are always complex because of constant dynamic interactions among many indigenous and extraneous factors which are associated with the microenvironment [111,121]. The dynamic interactions result in the changes in cellular morphology along with functional alteration or variation in cell–cell or cell–matrix interactions, maintain the homeostasis for cells which are associated with their communities [121]. When a normal condition transits to an altered condition, all the cells associated with the environment have to alter their functions to help to manage their survivability until a normal condition returns, or they have to go through the direction they receive to adapt to the altered condition, or they die if they cannot tolerate or fight against the abnormal situation. Breast cancer cell lines, which tend to aggregate, can form tumoroids. Tumoroids represent moderate tissue complexity as compared with the high complexity of native tissue. They facilitate the study of cell–cell or cell–matrix interactions and the analysis of tumor formation and progression towards cancer. It is also known that some subpopulations of tumor cells can represent cancer stem cells (CSCs) or tumor-initiating cells (TICs) that have stem cell-like features for tumor initiation and growth [112]. During tumoroid sphere formation, CSCs plays a significant role in the division and synthesis of ECM protein that supports cell–matrix interactions and acts as a scaffolding bed for dividing cells. Tirino et al. showed that some tumor cells could lose their stemness during the culture period after their differentiation from CD133+ cells to CD133- cells [122,123], which ultimately led to the formation of a necrotic area inside the tumoroid [124]. It has been shown that tumoroids can maintain the stemness characteristic of tumor cells by suppressing the differentiation process [112]. Some interesting findings show that cancer cells (CD133-), which rarely form aggregates due to the lack of stemness characteristics, can regain their stem cell-like properties in tumoroid or 3D culture systems [125,126]. A dynamic model provides a suitable relocalization opportunity for cells that trigger transformation of inactive cells to active stem-like cells [126,127]. Cell–matrix and cell–cell interactions build up a cohesive force that brings the cells close together, forming compact and smaller tumoroids [128]. Cells with high cohesive force are usually present towards the center of the tumoroids, while cells with less cohesive force present in the periphery of the tumoroids [128]. 

Although tumoroids can represent the complexity of tumors to a certain extent, the in vivo implantation of tumoroids extends the benefits of their study to help characterize cell–cell and cell–matrix interactions in physiological conditions. The study of how fibroblasts, macrophages, and some other inflammatory cells migrate towards a tumor microenvironment is not possible using the current available 3D culture systems such as tumoroids. Breast tumor growth and angiogenesis factors are progressive markers of tumor expansion in a tumor. Once tumoroids implant into subjects to generate xenografted tumors, the cancer stem cells that present in the periphery of the tumoroids actively adapt to the new environment, and represent the “reactive” cancer cells that start providing the microenvironment to promote tumor growth [129]. 

In the initial phase, the inflammatory response to tumoroids by the host immune system triggers an increment in CXCL1, a mediator of leukocyte influx recruiting neutrophil (CD45+ Neut+) [130]. The inflammatory phase ultimately transits to the tumor progression phase, arresting apoptosis and increasing cell proliferation similar to that of the wound healing process [131]. It is known that the fibroblasts especially present in the breast carcinoma produce the paracrine growth factors, ECM components, and proteolytic enzymes, as shown in Figure 3 [132]. A study has shown that stromal fibroblasts support cancer growth and promote tumorigenesis [133]. In the tumor environment, fibroblasts differentiate into CAFs; however, differentiation does not show similar patterns since it depends on the grades of neoplasticity of cancer cells [134]. CAFs also gain the ability to transform non-tumorigenic cancer cells to tumorigenic cancer cells, promoting cancer growth. They also boost the signaling cascades in the surrounding area of a tumor that help recruitment of stromal fibroblasts and other host cells for tumor progression [135]. During tumoroid transformation to a tumor in a host tissue, high expression of certain cytokines such as IL-6 and IL-8 regulates the immune and inflammatory responses and enhances the breast tumor growth [130,136]. The increased expression of IL-6 also supports migration of endothelial stem cells, and IL-8 aids the process of angiogenesis [130]. CAFs release stimuli factors, for example, TGF-β superfamily factors (FGF, HGF, etc.) that have the ability for EMT that is associated with E-cadherin loss, enhancing invasiveness of tumor cells for tumor progression [137]. CAFs, in addition, create an environment that helps to express more WNT ligands for tumor growth [138]. The matrix degrading enzymes (MMP-1, MMP-2, MMP-3, MMP-9, MMP-11, and MMP-14) secreted by CAFs breakdown the basement membrane barriers and play an essential role in EMT (Figure 3) [137,139]. MMP-1 itself has also been shown to promote tumor cell migration by activating a receptor, protease-activated receptor (PAR1), which is expressed in breast cancer cells [140].

Recently, CRISPR-Cas9 gene editing technologies are in practice to better understand breast tumor growth through specific mutagenesis of some mutation factors that are involved in malignant transformation [141,142]. This technology helps accurately knockout the genes of interest as well as shortens the culture time of fibroblasts [143]. Stefano et al. demonstrated CRISPR-Cas9-mediated somatic gene editing of mammary epithelial cells that caused E-cadherin loss and initiated invasive lobular breast carcinoma [144]. Since the mutation created by CRISPR-Cas9 was permanent, it was suitable to terminally differentiate cells and to efficiently express genes of interest in tumoroids [144,145]. When implanted into the mice, mutated tumoroid transforms into invasive cancer and mutated normal cells behave as cancer cells and proliferate rapidly for breast tumor growth [138]. 

Infiltration of fibroblasts into an implanted tumoroid supports complex ECM network formation after the deposition of laminin, fibronectin, hyaluronan, and glycosaminoglycans, etc. by fibroblasts [146,147]. Some of these components under the influence of tumor cells promote the migratory capacity of fibroblasts, for example, by increasing the extra domain A and B of fibronectin in the tumor environment [148,149]. Recently, Begum et al. further showed that CAFs promote directional cancer cell migration by aligning fibronectin through increased traction forces and contractility [150]. A tumoroid, therefore, enhances the early exponential growth phase in a xenografted tumor followed by a delay in growth as compared with a xenografted tumor from 2D cultures where early growth rate is slow due to the presence of both non-proliferating and necrotic cells, and even the total number of cells is higher [15]. CAFs also enhance cancer growth by increasing the secretion of stromal derived factor-1 (SDF-1) which directly stimulates the expression of cognate receptor, CXCR4, that initiates angiogenesis and tumor cell motility [20]. It is still under debate whether tumor cells transform fibroblasts to myofibroblasts for tumor growth or if the previously altered stromal microenvironment enhances the tumor growth [151,152]. Different populations of CAFs that are derived from several cell types may be present in a tumor with different overlapping or non-operating expressions of certain markers such as NG2, PDGFRβ, and αSMA [153,154]. CAFs organize the matrix with the help of a mediator, PDGFRα, which is also associated with connective tissue remodeling [150,155]. Organized matrix and integrin αvβ6 and α9β1 promote directional cell migration, facilitating tumor shape and its growth [150,156]. Further, formation of larger adhesions containing vinculin in CAFs, as compared with those in normal stromal fibroblasts, slows the turnover rate, increasing mechanical and traction forces that help to shape breast tumor growth [150,157]. 

## 5. Conclusions and Perspectives

Fibroblasts are heterogeneous with versatile functions that depend on their locations and purposes. On the one hand, they are essential to tissue repair, and on the other hand, they facilitate apoptosis, thereby, representing a totally opposite function for a fundamental purpose that is the maintenance of homeostasis and tissue integrity. Fibroblasts transform in a tumor microenvironment to activated CAFs, which are more active and proliferative, accelerating breast tumor growth under the direction of cancer cells. In addition, CAFs show the frequent random motility which is a type of migration with excessive expression of ECMs and other biological factors. CAFs can arrest apoptosis, thereby, facilitating uncontrolled abnormal growth. After tumoroid implantation, the cancer cells located on the surface of a tumoroid try to adapt in a new host environment. During this time, a tumoroid sensitizes local host tissue and evokes the inflammatory response. A tumoroid ultimately stabilizes its own microenvironment and transforms to a xenografted tumor by recruiting various host cells, for example, local fibroblasts. A tumoroid transforms local fibroblasts to CAFs that further enhance the inflammatory response which helps to recruit many inflammatory cells such as neutrophils and monocytes. Eventually, inflammatory cells that come into contact with a tumor turn into cancer-associated inflammatory cells. They proliferate rapidly, protect the tumor, and further accelerate migration of host cells towards the tumor as well as initiate new vessel formation in the growing tumor. 

Native tumor tissue mimicking a tumoroid supports the possibility of a benchmark to try to understand how fibroblasts migrate towards tumor tissue and take part actively in tumor formation. However, we are not there yet where we can monitor the migration of fibroblasts through an in vitro 3D system. We can only show the migration of fibroblasts to a tumoroid after xenografting and harvesting a tumor along with its surrounding host tissues in different time points or by using highly sophisticated in vivo live imaging systems that use contrast agents or a tumoroid of fluorescent tagged cells that help monitor tumor formation in real time. The 3D native mimicking substrata along with the systems, which could facilitate the flow of blood and tissue cells (also considered as biophysiological 3D dynamic culture system) as in a body system, would substantially help to demonstrate the role of fibroblasts in tumor formation, and to fully understand the mechanism of tumor growth. In addition, this system would make it easy to decipher the cancer cell signatures in an in vitro setup that would show expressions similar to in vivo tumor tissue. 

The recent advancement in a 3D system that uses a fluidic tumoroid culture to track tumor invasion is encouraging, suggesting that, in near future, there will be a fully functional tumoroid dynamic system that helps study cancer growth without the use of animals [9]. The delay in creating a biophysiological 3D dynamic culture system and incomplete information about tumor growth keeps us in the present conflicting situation about cancer growth mechanisms, and therefore makes the future timeline uncertain for successful personalized anticancer therapeutics.

## Data Availability

Not applicable.
